# Cadaver clots: a systematic review of the literature

**DOI:** 10.1007/s12024-025-00976-y

**Published:** 2025-03-03

**Authors:** Biagio Solarino, Laura Ambrosi, Marcello Benevento, Davide Ferorelli, Claas Buschmann, Simona Nicolì

**Affiliations:** 1 Section of Legal Medicine, Department of Interdisciplinary Medicine, University of Bari, Piazza Giulio Cesare 11, 70124 Bari, Italy; 2 Institute of Legal Medicine, University Hospital Schleswig-Holstein, Arnold-Heller-Str. 3, Building 28, 24105 Kiel, Germany

**Keywords:** Chicken fat clots, Agonal thrombi, Postmortem clots, Agonal period, Clots in forensic

## Abstract

Cadaveric blood is ubiquitous, and observed in various forms—liquid, coagulated, and clot-like—during autopsies. Understanding its state in postmortem vessels is essential for both scientific research and forensic investigations. Pulmonary thromboembolism (PT) is a leading cause of sudden death, often requiring medicolegal evaluation. While thrombus formation is primarily explained by Virchow’s triad, the distinction between antemortem, agonal, and postmortem clot (PMC) pathogenesis remains debated. This study aims to systematically review the literature to clarify the morphological and pathological differences among these entities in forensic practice. A systematic review of PubMed, Science Direct, Scopus, and Web of Science was conducted using predefined key terms: “clot,” “thrombus,” “chicken-fat,” “agonal,” “postmortem,” and “autopsy.” Articles were screened for relevance, and 11 studies meeting the inclusion criteria were analyzed. The review highlights a significant gap in comparative studies addressing antemortem versus postmortem clots. The literature lacks a consensus regarding their definitions, macroscopic and microscopic characteristics, pathogenesis, and relevance to determining the cause and timing of death. Existing studies present conflicting interpretations, limiting the reliability of forensic differentiation. The current understanding of antemortem, agonal, and postmortem clots remains incomplete. Our findings underscore the need for further research to establish standardized criteria for distinguishing clot types, which is crucial for forensic pathology and medicolegal evaluations.

## Introduction

Postmortem blood in humans is a ubiquitous finding, as it may be either wholly liquid, largely coagulated, or resembling clots or thrombi-like formations [1]. Distinguishing antemortem from agonal or postmortem clots is crucial for forensic pathologists as it has several medical and judicial implications [2]. Pulmonary thromboembolism (PT) is among the leading causes of sudden death, being traditionally one of the clinician’s big challenges due to the difficulty in diagnosis resulting from the non-specific signs or symptoms [3, 4]. PT typically results from deep venous thrombosis (DVT), originating in the pelvis or leg veins, etiologically caused by immobility due to natural diseases or traumatic injury that defines the manner of death. Hence, failure to diagnose pulmonary embolism or to administer antithrombotic prophylaxis may pose a professional liability issue for physicians involved in the diagnostic process of the disease [5]. Moreover, the presence of agonal blood clots in sudden or prolonged death at autopsy has relevant allegations in cases of homicide, intestacy rules in simultaneous death, and for establishing damage compensation for the patients dying after an agonal period [6].

**Fig. 1:**
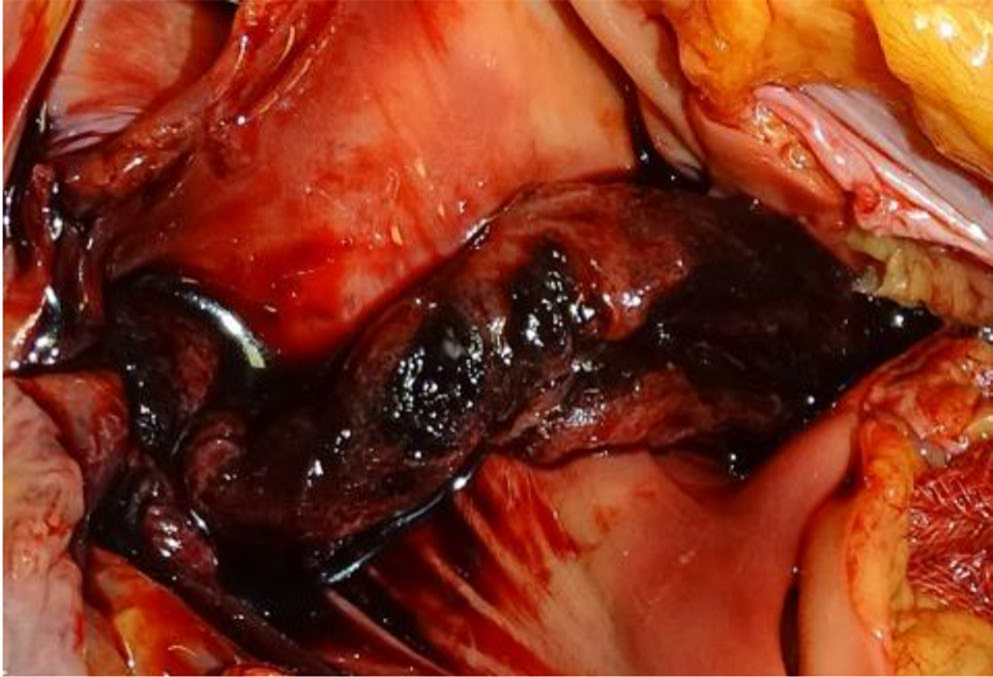
Typical “saddle” thrombus in a case of pulmonary thromboembolism, with typical granular surface, convoluted appearance and a pale tan to red-blue coloration

**Fig. 2:**
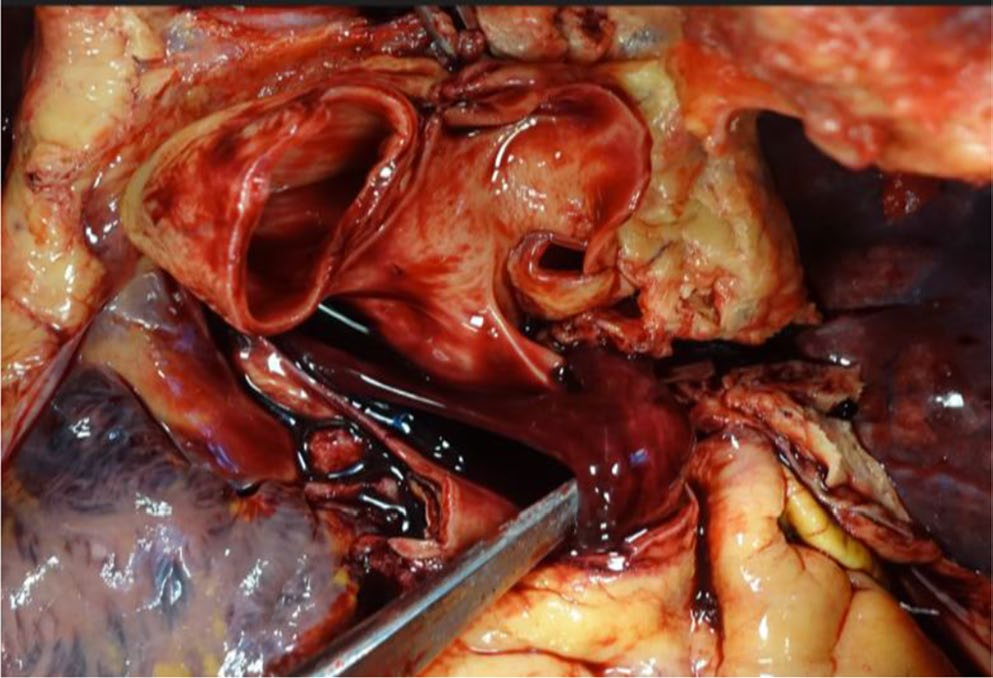
A chicken fat clot in situ, within the pulmonary artery. The smooth, shiny, non-convoluted surface is visible

**Fig. 3:**
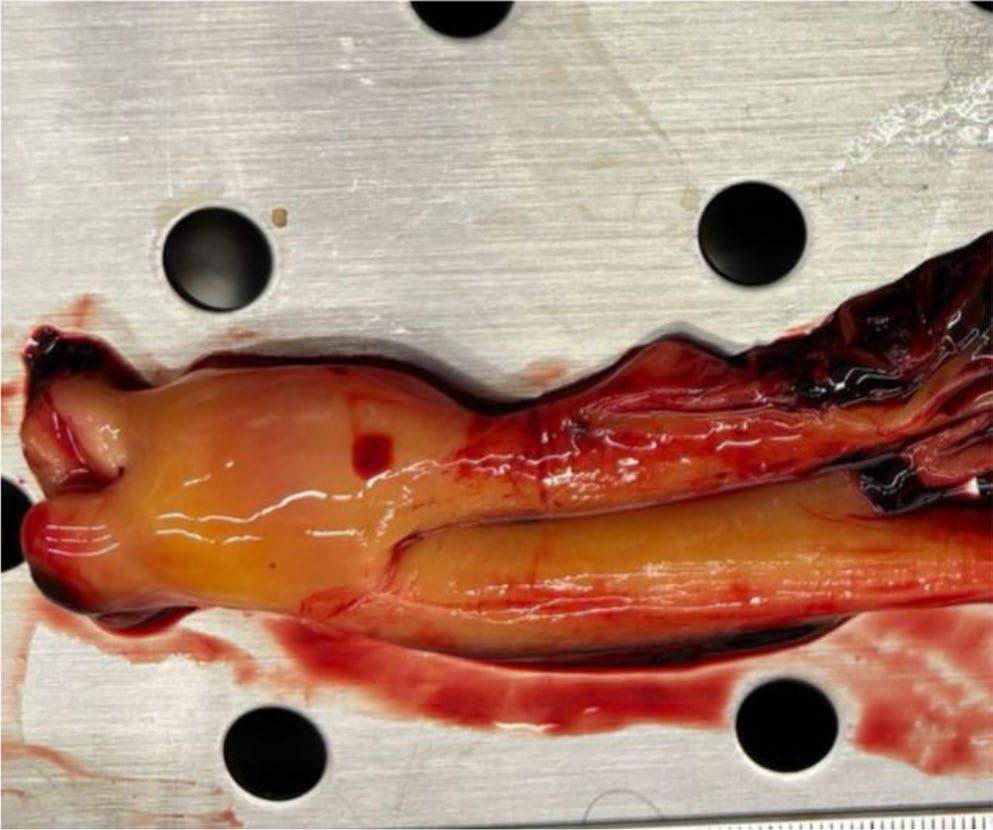
A chicken fat clot extracted from the vascular lumen. Its gelatinous consistency allows for complete removal (non-friable). The shine and regularity of its shape can be observed. The color varies from deep red to yellow due to fibrin accumula

**Fig. 4:**
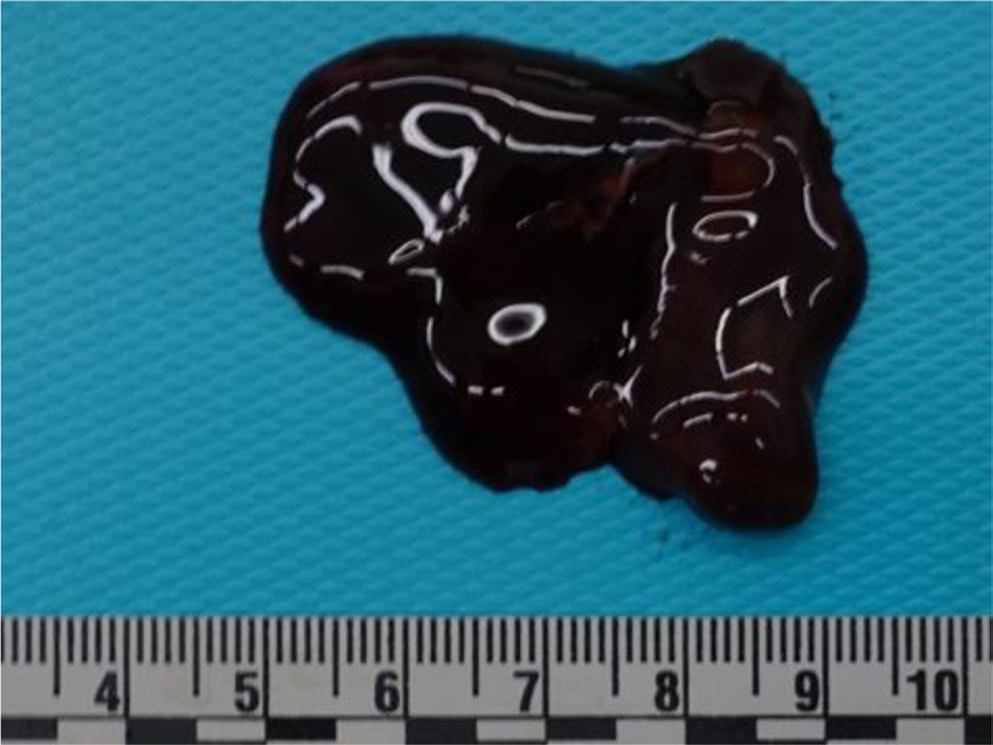
A red currant jelly clot (cruor) extracted from the vascular lumen of the pulmonary artery, with a typical soft and amorphous surface and friable consistency

The process of thrombus formation is primarily explained through three risk factors that characterize the so-called “Virchow’s triad” (Rudolf Virchow,1821–1902). Endothelial injury is the most important factor and can lead to alterations in local blood flow and hypercoagulability, predisposing to thrombosis [7, 8]. Accordingly, the gross features of antemortem vascular thrombi are well-known, especially the description in the pulmonary trunk as a saddle-entangled embolism, slightly adherent to the vessel with a pale tan to red-blue appearance is emblematic (Fig. 1) [9]. Nowadays, postmortem imaging is one of the most innovative features in forensic medicine. Postmortem computed tomography (pmCT) and enhanced Magnetic Resonance Imaging (MRI) are valid tools for differentiating antemortem from postmortem clots [10]. Microscopically, Zahn’s lines are commonly cited as pathognomonic features of antemortem thrombi (AMT), and some studies have emphasized the need to establish firm histological criteria for age determination of fatal venous thromboembolism [11–13]. Therefore, while gross and microscopic criteria for diagnosing AMT are routinely used in autopsy practice, identifying agonal or postmortem clots and their significance in forensic pathology remains a subject of ongoing debate and uncertainty. The so-called “chicken fat clots” (CFCs) are often found in the heart and large vessels, but they are typically non-adherent to the underlying lumen (Figs. 2 and 3). They appear as gelatinous substances with a typically yellowish or pale-yellow surface, the supernatant portion of coagulated clear plasma (chicken fat) overlying a portion of settled red blood cells. Some studies have stated that chicken fat clots are produced postmortem, while other authors consider them as agonal thrombi that might be distinguished from postmortem red current jelly clots (cruor), which have soft and amorphous surfaces (Fig. 4) and may be seen both in the heart and systematic circulation [8, 14, 15]. The mechanism underlying their formation and the time of formation relative to death remain unclear in both agonal and postmortem clots. Table 1 summarizes the main characteristics of CFC, cruor, and thrombi.

**Table 1:** Summary table of the key features of thrombi, chicken fat clots, and cruor

	Thrombi	Chicken Fat Clot	Cruor
Macroscopic appearance	Granular surfaces; variable white-tan to red coloration; generally spiralized; tubular or cylindrical when uncoiled	Fine granular to smooth glistening surfaces; translucent yellowish/pale yellow component (supernatant) and red coagulated portion	Gelatinous, bright dark red, amorphous, smooth surfaces
Texture/consistency	Dry and friable	Variable soft to mild elasticity	Soft, falls apart on handling
Composition	Cut sections show coarse, pale gray stripes at the periphery of the thrombus (fibrin and leukocytes), while the core was mostly dark red (red cell)	The translucent portion contains mainly fibrin and leukocytes; the coagulated portion is a uniform mixture of red blood cells, leukocytes, and fibrin.	Aggregated erythrocytes with scattered individual leukocytes and few dispersed platelets and strands of fibrin
Location	Generally in venous circulation; the portions that break off (emboli) follow the blood flow typically stopping in pulmonary vessels	Right side of the heart (pectinate muscles) and systemic circulation	Venous and systemic circulation

We conducted a comprehensive systematic review of PubMed, Science Direct, Scopus, and Web of Science databases to gather insights into the differentiation among antemortem, agonal, and postmortem clots during daily autopsy practice and to investigate what has been written in the last twenty years if these questions still arouse interest. The aim was to investigate the literature on clots in-depth, referred to as “postmortem clots” or “chicken fat clots”, identifying commonly used study techniques and exploring any possible correlations between clots and agonal intervals. 11 articles were included based on predefined key strings: “clot”, “thrombus”, “chicken-fat”, “agonal”, “postmortem”, and “autopsy”. Our findings reveal that few studies have thoroughly compared ante- and perimortem clots, and the available literature lacks consensus regarding their definitions, macroscopic and microscopic characteristics, pathogenesis, and relationship to the cause and timing of death. Likewise, our review underscores the need to improve research in this area.

## Methods

### Search strategy

This systematic review followed the Preferred Reporting Items for Systematic Review and Meta-Analyses (PRISMA) guidelines [16].

We used a systematic literature search from PubMed, Science Direct Scopus, and Web of Science databases on January 5, 2024, using the following strings: (“clot”; OR “thromb*”; OR “chicken fat”; OR “chicken-fat” ) AND ( “agon*” OR “postmortem”; OR “post mortem”; OR “post-mortem”; OR “surviv*”; OR “terminal”; ) AND ( “autop*”; OR “forensic*”) in the title, abstract, and keywords, and selected works published between 2003 and 2023.

One investigator (MB) conducted the selection process and extracted the data independently with PRISMA standards of two-times repetition to minimize study bias. Three investigators (SN, LA, and BS) verified the data, and the fourth investigator (CB) validated the methodology for the qualitative synthesis.

### Inclusion and exclusion criteria

The inclusion criteria for eligibility were: (1) focus on a detailed description of clots (gross, microscopic, or imaging), (2) detection of clots in major vessels or heart chambers, microthrombi were excluded (3), reviews were excluded (4).

All articles’ references were screened, reviewed, and cross-checked for relevant studies. Only papers in English and Italian were selected for this study.

### Data extraction

The identified papers were screened independently by two reviewers in duplicate, based on their titles and abstracts, and papers that met the inclusion criteria outlined above were assessed for eligibility by full-text reading. Articles whose titles and abstracts were uncertain were also included. Discrepancies were resolved by consulting a third opinion or conducting Delphi rounds with all the authors when necessary. The review protocol was registered with Open Science Framework (registration doi: 10.17605/OSF.IO/PS9JM).

We used a standardized form to extract data from the included studies to assist in study quality and evidence synthesis. Extracted information included the following: (1) aim of the study, (2) cadaver’s description, (3) blood clot location and collection number, (4) blood clot’s conservation and preparation techniques, (5) type of analysis, (6) blood clot description, (7) description of agonal period where applicable, (8) comparison with thrombi where applicable and (9) main results of the study.

Data extraction was performed by three reviewers independently and in duplicate. A fourth reviewer was consulted when necessary.

### Quality assessment

Three independent reviewers performed a quality assessment to rate the quality of the selected studies. The quality assessment tool was drawn directly from https://jbi.global/critical-appraisal-tools. JBI offers a different assessment tool, with a variable number of questions, depending on the type of study. The tools consisted of eight to eleven questions, each of which can be answered with “yes,” “no,” “unclear,” or “not applicable,” with a final “overall appraisal” judgment which includes the following alternatives: “Include”, “Exclude”, “Seek further information”. The possibility of a short comment justifying any exclusion is also included.

## Results

**Fig. 5:**
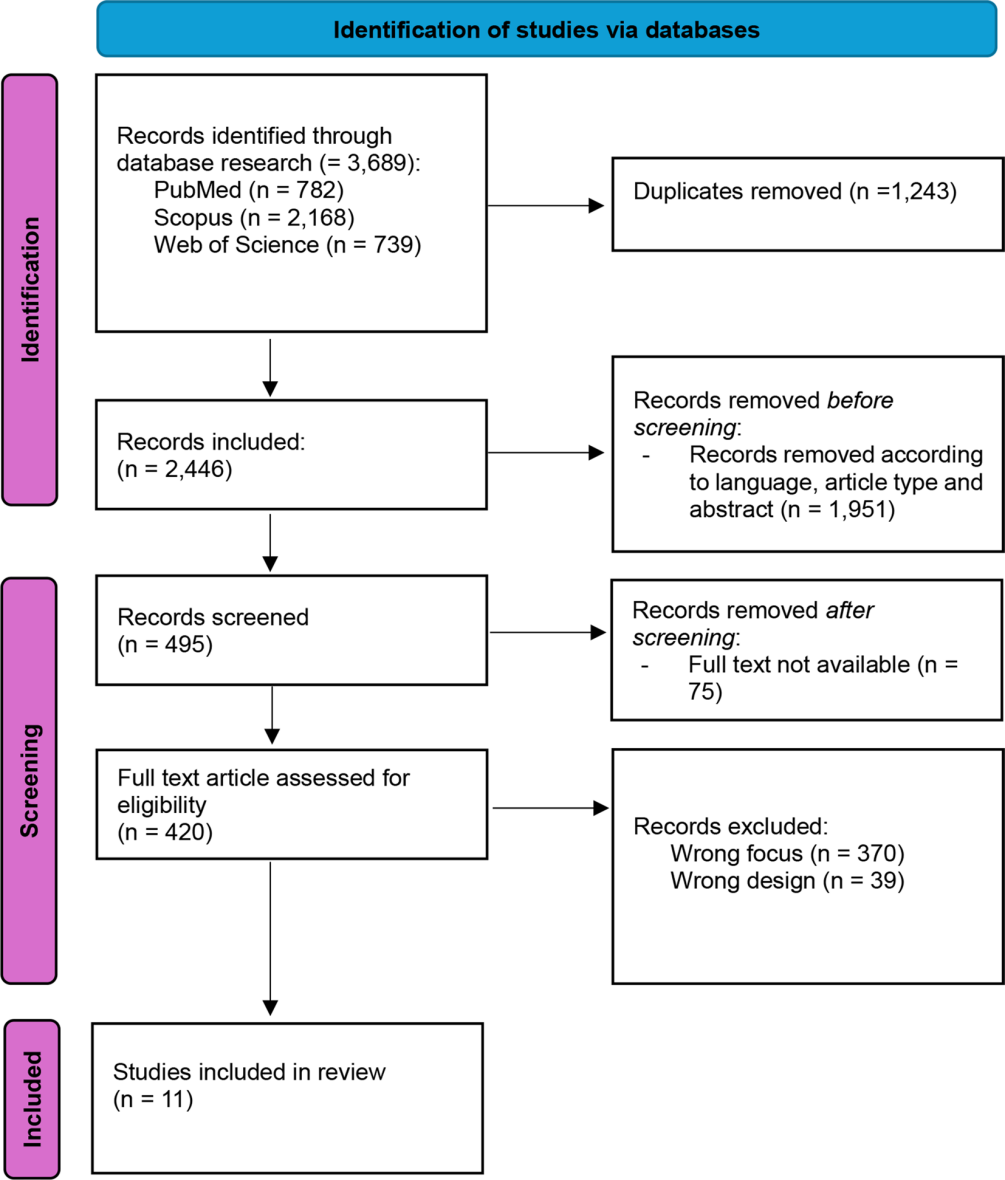
Flow diagram of paper selection

We identified 3,689 articles. The duplicated articles (*n* = 1,243) were removed from the list. The remaining 2,446 papers including the titles and abstracts were screened, resulting in 448 articles for further analysis. A full-text review was carried out to exclude non-eligible articles, thus generating 11 references for qualitative analysis. These 11 eligible references were carefully assessed following the aim of the systematic review. We present the PRISMA flowchart in Fig. 5 and the literature demography in Table 2. The quality assessment is available in the Supplementary material. All studies subjected to the tool were included in the review, having been judged scientifically valid.

Of the 11 articles selected, six focus on applying postmortem diagnostic imaging to evaluate and assess the feasibility of differential diagnosis using these methods, primarily for PT [17–22]. Two articles investigate the formation mechanisms of CFC and their correlation with the agonal interval [23, 24]. Two others aim to define the histological characteristics of AMT and postmortem clots [15, 25]. Finally, one article examines the relationship between alcohol intoxication and the presence of clotted blood in the central vessels of cadavers [26]. The findings of the articles cited above will be summarized below.

**Table 2:** Literature demography

Author (Year)	Country	Study Design	Population	Cadaver description	Type of analysis	References and characteristics of agonal period
Matsuda et al. (2023)	Japan	Observational cross sectional	25 cadavers	Causes of death: 6 traumatic, 5 infectious diseases, 2 central nervous system hemorrhage, 7 drowning, 5 others. Postmortem interval < 7 days; age 27–91.	Immunohistochemical (antibodies against neutrophil elastase, myeloperoxidase, histone H2A, H2B, and H4)	Not specified
Krywanczyk et al. (2023)	USA	Observational cross sectional	50 cadavers. 50 arterial and 50 venous surgical thrombectomy specimens collected after removal by thrombectomy or embolectomy	50 autopsies – Causes of death: 20 infections, 10 cardiovascular or cerebrovascular diseases, 8 malignancies, 3 surgical complications, 2 interstitial lung diseases, 7 others. 26 men and 24 women, average 63.1 years. Average postmortem interval: 36.06 h.	Macroscopic examination Microscopic examination immunohistochemical examination	Not applicable
Flach et al. (2023)	Switzerland	Observational cross-sectional	113 cadavers	Causes of death: 88 natural deaths, 18 accidents, 1 suicide; 1 case with complications during surgical intervention; 5 unclear cases. The overall postmortem interval from time of death to pmMRI scan ranged from 2 to 168 h (mean 31.6 h). The overall postmortem interval from time of death to autopsy ranged from 4 to 190 h (mean 41.6 h).	Macroscopic examination. Microscopic examination (hematoxylin and eosin + Elastin van Gieson); Imaging techniques	Not applicable
Kasagawa et al. (2021)	Japan	Observational cross sectional	112 cadavers of 150 consecutive cases	72 men, 40 women, mean age 61 years, range 3–6 years. 34 cases involving putrefaction, 3 loss of blood in the heart due to fatal bleeding, and 1 metal artifact were excluded.	Imaging techniques	Assessment performed by a forensic expert but not specified
Ampanozi et al. (2016)	Switzerland	Observational cross-sectional	31 cadavers	12 cases with autopsy confirmed PT as the cause of death and available pmCT, 19 cases with autopsy and pmCT that cause of death were other than PT. Cases with advanced decomposition, amputation of at least one lower extremity, fractures of the lower extremities, non-parallel positioned legs on the CT couch that exceeded the field of view, or medical emergency infusions at the lower extremities were excluded.	pmCT	Not applicable
Hansma et al. (2015)	USA	Observational cross-sectional	238 cadavers	Causes of death: 80 cases of rapid sudden death by violence, 21 cases of death caused or complicated by “true” pulmonary emboli.	Macroscopic examination Microscopic examination	Not specified
Burke et al. (2014)	Australia	Case ser**i**es	13 cadavers	Causes of death: suspected massive pulmonary thromboembolism with postmortem CT pulmonary angiography; 6 male, 7 female	Imaging techniques (postmortem CT pulmonary angiography)	Not applicable
Jackowski et al.(2012)	Switzerland	Case series	8 cadavers	8 autopsies of sudden death without medical history underwent pmMR before autopsy	Imaging technique	Not applicable
Uekita et al. (2007)	Japan	Observational cross-sectional	53 cadavers	Survival time: 20 cases survived less than 3 h. 17 cases survived more than 1 day. 16 cases died of inflammatory diseases, chronic heart failure, hemorrhagic shock, drowning, or other cases survived more than 1 day except drowning (survived less than 0.5 h), phenobarbital poisoning (survived less than 12 h), and hemorrhagic shock (survived less than 1 day).	Microscopic examination	Not specified how survival time was measured
Fracasso et al. (2007)	Germany	Observational cross-sectional	138 cadavers	All asphyxial deaths. Excluded asphyxial deaths with signs of putrefaction and Blood Alcohol Level (BAL) > 1‰.; 69 asphyxial deaths with BAL > 1‰.; 69 asphyxial death with BAL of 0‰.	Macroscopic examination	Not applicable
Jackowski et al. (2006)	Switzerland	Cross-Sectional	44 cadavers	Not specified	Gross examination (only photo) Imaging techniques (pmMRI)	Not applicable

pmMRI: post-mortem magnetic resonance imaging; pmCT: post-mortem computed tomography

### CFC and agonal period

Uekita et al. examined CFC samples from 53 autopsies, classifying cases based on age, gender, weight, height, blood alcohol level (BAL), cause of death, survival, and postmortem interval [23]. The samples were treated with rabbit polyclonal antibodies against fibrin and hematoxylin-eosin. Three distinct patterns of the arrangement were identified: wavelike fibrin fibers, short fibrin fibers, and a mixed pattern. The results revealed a correlation between the wavelike fibrin fibers pattern and a survival interval of less than 3 h. Conversely, the short fibrin fibers pattern was associated with a survival interval exceeding 1 day, often linked to causes of death such as malignant tumors or acute and chronic inflammatory pathologies. The main limitation of this study appears to lie in the definition of survival interval, which, in the authors’ opinion, was determined in a somewhat generic manner, relying heavily on the cause and circumstances of death. While the fatal event is relatively straightforward to define in cases of burns, suffocation, hemorrhage, or witnessed sudden cardiac deaths, it becomes more complex in conditions such as bronchopneumonia, malignant melanoma, chronic heart failure, and other typically chronic diseases included in the study.

A second and most recent study by Matsuda et al. (2023) proposes the hypothesis that neutrophils can play an important role in CFC pathogenesis by forming extracellular networks (NETs) [24]. The immunohistochemical study utilized antibodies against neutrophil elastase, myeloperoxidase, and histones. The concentration of ethanol in blood and urine and the blood c-reactive protein values were analyzed, and a state of disseminated intravascular coagulation (DIC) was also found. The results confirmed that NETs formation is also evident in CFC, not only in deaths following a longer agonal period but also in sudden deaths. Based on these findings, the study hypothesizes that CFC formation should be related to antemortem infectious or inflammatory conditions. The platelet contributions to the formation of NETs and deep vein thrombosis during infections or inflammatory disease were demonstrated in people who died of COVID-19 [27]. A weakness of Matsuda’s study can be found in the explanation proposed for those cases of sudden death with evidence of CFC, where the high number of neutrophils is hypothesized to be correlated to factors that stimulate neutrophil recruitment (hormonal stimulating factors or even physical exercise), although these remain speculative and unconfirmed. The study highlights the lack of a specific definition for the agonal interval (“survival time”). For instance, questions arise about how to define the survival interval in cases of pneumonia or when determining the onset of the fatal event in a case with “senility” as the cause of death.

Some references to the agonal period, in addition to the studies by Uekita et al. and Matsuda et al. [23, 24], are also found in the publications by Kasagawa et al. (2021) and Hansma et al. (2015). Kasagawa’s study, which will be examined in greater detail below, correlates specific areas of hyperdensity detected by pmCT (later confirmed as clots during autopsy) to the manner of death. The study divides the death process into “slow” and “rapid”, with a determination made by a forensic expert in the absence of additional details [21]. Hansma et al. analyzed a total of 238 autopsies, distinguishing cases between rapid and violent deaths (e.g. traffic accidents, gunshot wounds) and deaths caused or complicated by pulmonary embolisms. However, these are not detailed about the agonal period [15].

### Imaging techniques for clots or thrombi detection

A pioneering study was published by Jackowski et al. in 2006 to describe the changes in blood and the postmortal phenomena starting from the moment of interruption of the circulation: sedimentation, coagulation, and livor formation studied using MRI and multi-slice computed tomography (MSCT) [17]. Forty-four corpses were analyzed; the coagulation phenomenon was found in 14 cases. In all cases, clots were observed within the right atrium or pulmonary artery, while only 10 were clots located in the left chambers or the aorta. Jackowski specifies that rapid coagulation, compared to slow sedimentation, envelops the erythrocytes before sedimentation begins, leading to a homogeneous blood clot. On the contrary, slow coagulation and rapid sedimentation, for example, in pre-mortal inflammatory conditions, divide the blood clot into more signal-intensive with less signal intensity on T2-weighted images. The sedimentation, typically postmortal, makes it easy to distinguish what is postmortal from what occurs in an active cycle with this method.

Jackowski continued his studies in the field, using 3-T MR for the differential diagnosis between PT and postmortem clots, managing to describe a difference in signal and intra-vascular disposition of the two entities using pmMRI, although on a tiny sample indeed [18].

In 2014, Burke et al. analyzed 13 cases with strong clinical suspicion of death due to PT, which was followed by autopsy confirmation using pmCT Pulmonary Angiography. The images obtained were studied according to precise criteria: the absence or presence of symmetric filling of the segments of the pulmonary arteries by the contrast; the presence or absence of modest filling defects of the lumen, the continuous filling defect of the contrast medium from the right ventricle to the pulmonary outflow tract up to the two pulmonary arteries, the latter strongly suggestive of a postmortem clot [19]. Similarly, Ampanozi et al. in 2016 tried to identify possible characteristic patterns in cases of PT of signs of thrombosis of the lower extremities and again through the description of the pulmonary trunk and main pulmonary artery branches in unenhanced pmCT by retrospective analysis [20]. The study by Kasagawa et al. (2021), which has already been partly mentioned, aims to classify the types of hyperintense areas in the cardiac cavities via pmCT and compare them with the autopsy results, following the hypothesis that the appearance of blood clots in pmCT may provide important clues as to the cause and manner of death. Hyperdense areas in the heart cavities were classified into four types based on their shape, resulting in block-like and cast-like hyperdense areas in the right heart cavities being predictive of blood clots in the heart cavities in forensic autopsies [21].

The latest article by Flach et al.aimed to diagnose PT using pmMR. Researchers prospectively examined 113 subjects to identify PT and postmortem clotting (cruor). PT was detected in 20 cases, while the remaining 93 cases were analyzed for cruor morphology. Age grading of thrombi was performed using pmMR, autopsy, and histology. What we consider most interesting, for the focus of this review, concerns the description of the cruor [22]. Flach et al., referencing earlier studies, assert that within the same postmortem blood clot (cruor), two distinct portions form due to the sedimentation of blood components. The ‘red currant jelly clot’ portion is rich in erythrocytes and described as darker, with a livid red color, smooth surface, and glossy appearance. This portion forms more rapidly and typically results from the stratification of red blood cells during postmortem coagulation.

On the other hand, the CFC portion is poor in erythrocytes and is composed primarily of fibrin, leukocytes, and plasma. It appears lighter in color, with grayish or yellowish tones, and also has a smooth and glossy surface. This portion forms more slowly than the ‘red currant jelly’ component. Both portions coexist within the same postmortem clot and exhibit a clear horizontal separation caused by the gravitational settling of blood components in the absence of body movement. In this case, some unclear passages emerge again: if for some authors the “cruor” are types of clots different from CFC, for other authors, as in this case, the two entities coexist in the same clot.

### Histological peculiarities of antemortem and postmortem clots

Hansma et al. (2015) analyzed 238 autopsies, of which 80 were rapid deaths and 21 cases complicated by PT [15]. The so-called cruor (postmortem blood clots typical of venous vessels) were present in all cases analyzed. Agonal thrombi were observed in 122 cases (89% of cases of slow death). No agonal thrombi were observed in cases of rapid deaths. Postmortem clots were defined according to the following macroscopic features: the so-called “red currant jelly clots”, red mostly present in arterial vessels, to be distinguished from “black currant jelly clots” or “cruor” present in venous vessels. Agonal thrombi, on the other hand, have a variable surface from fine granular to smooth, do not fill the lumen of the vessels, have a variable color from yellow to red, and are often found in the right chambers of the heart. Hansma considered it proper to report the presence of postmortem clots in autopsies, as they were considered helpful in excluding specific causes of death.

Krywanczyk et al. (2023) focused on the gross and microscopic features that distinguished true antemortem thrombus from artifactual postmortem clots, starting from an update of Zahn’s lines [25]. The authors reasoned that Zahn’s lines consisted of serpiginous bands formed by nests of platelets wrapped in fibrin. Though such findings are cited as a pathognomonic feature of AMT, the paper underscores no unanimity on their definition, not even on their nature, whether macroscopic or microscopic. The study analyzes 50 histological samples of postmortem clots, 50 samples of arterial thrombi, and 50 of venous thrombi obtained via thrombectomy or embolectomy. The description of the formations is both macroscopic and microscopic, finally leading to the identification of some characteristics that differentiate postmortem clots (distribution pattern of CD61, absence of neutrophilic karyorrhexis, presence of bone marrow elements) from antemortem thrombi (thick bands of platelets wrapped by fibrin, geographic pattern of CD61, diffuse neutrophil karyorrhexis).

Although it cannot be included in the previous topics, the study by Fracasso et al. (2008) seemed interesting because it was cited several times in the publications included in this review. In our opinion, the peculiarity concerns the description of a type of clots not attributable to CFC but, at the same time, provides an interesting correlation with blood alcohol levels [26]. Fracasso et al. examined 138 autopsies that were divided into two groups: 69 asphyxial deaths with BAL level > 1‰ and 69 asphyxial deaths with BAL of 0,00‰. From these autopsies, the authors reported the degree of blood coagulation in the central vessels, identifying three different degrees of coagulation. The study’s conclusions demonstrate how a distinctly positive BAL is often associated with an important blood coagulation state.

## Discussion

The definition of chicken fat clots originates from the American Pocket Medical Dictionary (1898) by William Alexander Newman Dorland, who describes it as a yellowish blood clot. In German forensic literature, such formations are described as “Speckhautgerinnsel,” a bacon derm-like clot [28, 29]. All the authors, including the most authoritative forensic and pathology textbooks, seem to be considering CFC and bacon derm-like clots as postmortem findings [14, 30, 31].

The primary aim of this systematic review was to emphasize the knowledge of this material for forensic pathologists who face clots in vessels or cavities during daily routine autopsy. When an entangled thrombo-embolus is found saddling the pulmonary trunk and within the main pulmonary arteries and their branches, arising from thrombi in the lower leg’s veins, all pathologists agree about the pathogenesis of such antemortem formations [32]. In these cases, clots’ macroscopic features, clinical history, laboratory, and inherited risk factors for thrombophilia are often enough to define their nature. In such circumstances, postmortem radiology and routine histology assessment, including immunohistochemical methods, can be used to confirm the cause and manner of death and to determine the approximate age of fatal PT [33, 34]. PT represents one of the leading manifestations of sudden death and implies lots of medicolegal issues. On the contrary, investigating the difference between agonal and postmortem clots is still challenging.

Despite the deep analysis of the studies reported, the evidence about postmortem clots remains unresolved. What happens when a human body transits from life to death? In common practice, observing yellowish clots in the pulmonary arteries, other large vessels, or heart chambers, is roughly alleged to be a sign of a prolonged dying period. However, understanding the pathophysiology mechanism of the blood in the corpse before or after death could be of vital importance from scientific and judicial inferences. Sure, prolonged death accounts for a clear definition of an agonal period. Many interesting papers are geared primarily toward the role of biochemical markers for evaluating the dying period (ultrashort, short, long ) that may be initiated by disease or trauma [35–39]. More unclear is the role of CFCs, which, in the revised literature, is considered either agonal or postmortem clots, and a primary issue remains: can blood coagulate postmortem in stationary flow? Why did some cadavers show different features of coagula (yellowish, red cruor) while others are entirely liquid? How can the mode of death influence the blood’s condition after dying? The Virchow triad’s most important factor is endothelial injury. According to the valve cusp hypoxia hypothesis (VCHH), the endothelium valve cusp leaflets were damaged by circulatory failure due to several diseases, including sepsis [40]. This progressive systemic blood deoxygenation induces white cells to repair dying vascular endothelium, which releases significant amounts of procoagulant factors or may synthesize less anticoagulant effectors [8]. Blood flow turbulence contributes to clotting events by forming stasis. That is the reason why pathologists must describe every form of clot or coagula in the cadaver, along with organ congestion, for a better understanding of the cause and time of death.

Matsuda et al. pointed out that thrombus formation was due to a congregation of neutrophils, fibrin, platelets, and erythrocytes closely related to vascular endothelial dysfunction [24]. According to VCHH, the entity of neutrophils/platelets depends on the cells viable near the dead endothelium. If death is sudden, as it happens in acute respiratory failure, blood cells die also rapidly, and thrombi cannot form. This theory is in agreement with fluid and dark de-oxygenated blood reported in asphyxia death. Furthermore, in gradual circulatory failure, endothelium damage resulting from hypoxia allows neighboured white cells and platelets to congregate throughout the vascular system, forming clot-like thrombi. That is the possible reason we found clots also in cases of sudden death in which it is mandatory to verify possible antemortem infectious or inflammatory conditions, according to Matsuda’s hypothesis. In this latter condition of under-perfusion, we found most CFCs in the right heart and pulmonary arteries because they suffered more than the rest of the circulatory system due to endothelial hypoxia [40].

Hence, since thrombosis is a pathophysiological response to endothelium damage, it is hard to think that clots form after death without active blood circulation. None of the authors that classified CFCs as postmortem findings can explain their pathogenesis. Kriwanczyk et al. compared histological features of antemortem to postmortem clots [25]. However, they assume the passive activations of platelets and neutrophils occurred at the very end of life (agony? ) and not after death. Matsuda et al. remark that CFCs are more likely to be detected in cadavers with chronic inflammatory disease or when an acute microbial invasion stimulates innate response, increasing levels of chromatin and activating blood coagulation [24]. Fulfilling these complex processes that involve a balance with coagulation and fibrinolysis is more suitable to start before than after death. Moreover, when cardiovascular circulation stopped after death, postmortem like-coagulation was due to the gravitational pooling of blood (like curdled milk) into capillaries within the dermis, explaining the livor mortis phenomenon [41].

Thomsen and Krisch studied such a phenomenon and explored the possibility of activating platelets after death to demonstrate that blood can coagulate postmortem. Fluid blood was taken from cadavers and left at room temperature until spontaneous lysis [42]. Despite the study clearly showing that platelets in cadaver blood were not activated, their findings were cited in articles supporting the existence of postmortem thrombi [43]. If fibrin polymerization occurred before death, it is possible to observe platelet activation, which continues after death. According to this evidence, the authors who denied the possibility that clots, coagula, or thrombus could form in corpses after death accept the hypothesis that they begin forming as the patient is dying and continue to form postmortem. Such a phenomenon could be following supravital reactions and/or residually oxygenated blood for a period after the circulation has stopped. This statement follows the theory of Rigor mortis formation. Vital coagulation requires ATP that terminates after death but is still generated by anaerobic glycolysis for a short period postmortem [44].

If the question regarding the pathogenesis of cadaver clots remains unsolved, some difficulties arise also in distinguishing gross and histopathological features.

Knight points out that antemortem thrombo-emboli are readily recognizable and can be easily distinguished from postmortem clots [14]. However, if red cruor is unanimously deemed a reddish-purple mass of erythrocytes, the different components of platelets and white cells in clots and emboli suggest being cautious in the gross differentiation. Hence, Kriwanczyk stated that none of the macroscopic features are absolute, and pathologists must be careful of pitfalls.

Histopathology is alleged to be the gold standard exam in distinguishing cadaver blood clots. Microscopically, Zahn’s lines are seen as hallmarks of early venous thrombi. Recent studies, however, have demonstrated that this attribute has no absolute scientific accuracy for a series of reasons. First, no studies microscopically compare postmortem emboli/thrombi and clots, and the main issue regards the lack of an unequivocal definition of Zahn’s lines [25]. The layers of fibrin and platelets with erythrocytes and leukocytes are visible to an expert’s naked eye. However, in literature, the authors used this definition also when observing alternating bands of platelets and red blood cells, amorphous masses of fibrin and platelets, thin fibrin strands among large aggregates of erythrocytes and linear markings on the surface of thrombi [45–47]. Moreover, that is why it is not enough to examine clot or thrombi specimens microscopically; specific skills are mandatory.

## Conclusion

The state of the blood in postmortem involves lots of medicolegal issues. This systematic review identified all existing evidence on cadaver clots and likewise showed difficulties distinguishing gross, histological, and imaging features between antemortem from agonal and postmortem clots. Our findings underscore the need to improve understanding of the pathogenesis of clots faced in daily autopsy practice. Studies such as those conducted by Matsuda and Krywanczyk can act as catalysts towards the topic, and open up new research ideas that can bring us closer to understanding the pathogenesis of CFC and, therefore, to a standardization of their study. Hence, we recommend further studies focusing on the role of specific factors, including preexisting diseases or mode and time of death associated with chicken fat clots. Such research can also improve our thoughts in the agonal period.

## Key points

1. Distinguishing antemortem from “agonal” clots is crucial for forensic pathologists as it has several medical and judicial implications.
2. The mechanism underlying the formation and the time of antemortem and perimortem clots remain unclear in agonal clots.
3. The comprehensive systematic review demonstrates that the pathogenesis of cadaver clots remains unsolved, and some difficulties arise in distinguishing gross and histopathological features in forensic practice. Microscopic examination of clots is not enough; specific histopathology skills are mandatory.
